# Breastfeeding, lung volumes and alveolar size at school-age

**DOI:** 10.1136/bmjresp-2015-000081

**Published:** 2015-07-06

**Authors:** Cristian M Dogaru, Manjith Narayanan, Ben D Spycher, Anina M Pescatore, John Owers-Bradley, Caroline S Beardsmore, Michael Silverman, Claudia E Kuehni

**Affiliations:** 1 Institute of Social and Preventive Medicine, University of Bern, Switzerland; 2 Department of Children, Young People and Education, University Campus Suffolk, UK; 3 Division of Child Health, Department of Infection, Immunity & Inflammation, University of Leicester, UK; 4 School of Physics and Astronomy, University of Nottingham, UK

**Keywords:** Paediatric asthma

## Abstract

**Background:**

Previous studies found larger lung volumes at school-age in formerly breastfed children, with some studies suggesting an effect modification by maternal asthma. We wanted to explore this further in children who had undergone extensive lung function testing. The current study aimed to assess whether breastfeeding was associated with larger lung volumes and, if so, whether all compartments were affected. We also assessed association of breastfeeding with apparent diffusion coefficient (ADC), which measures freedom of gas diffusion in alveolar-acinar compartments and is a surrogate of alveolar dimensions. Additionally, we assessed whether these effects were modified by maternal asthma.

**Methods:**

We analysed data from 111 children and young adults aged 11–21 years, who had participated in detailed lung function testing, including spirometry, plethysmography and measurement of ADC of ^3^Helium (^3^He) by MR. Information on breastfeeding came from questionnaires applied in early childhood (age 1–4 years). We determined the association between breastfeeding and these measurements using linear regression, controlling for potential confounders.

**Results:**

We did not find significant evidence for an association between duration of breastfeeding and lung volumes or alveolar dimensions in the entire sample. In breastfed children of mothers with asthma, we observed larger lung volumes and larger average alveolar size than in non-breastfed children, but the differences did not reach significance levels.

**Conclusions:**

Confirmation of effects of breastfeeding on lung volumes would have important implications for public health. Further investigations with larger sample sizes are warranted.

**Key messages**

- This study is the first to report data on the possible association between breastfeeding and alveolar size.
- While the study does not provide strog evidence of an association, it suggests that breastfeeding may be associated with increased lung volumes and alveolar size, particularly in children of mothers with asthma.

## Introduction

Breastfeeding has many beneficial effects for children and mothers.^1^ The impact on respiratory health is, however, less clear. Studies have shown that breastfed children have fewer and less severe respiratory infections than their non-breastfed peers.^2–5^ The influence of breastfeeding on lung development has been investigated by few researchers, with mixed results.^6–11^ Most studies reported larger normalised lung volumes in breastfed children, usually higher forced vital capacity (FVC) or forced expiratory volume at 1 s (FEV_1_).^6^ ^8–11^ In a recent study, we found that children breastfed over 3 months had higher forced mid-expiratory flows (FEF_50_) at school-age.^12^ There was also evidence of an effect modification by maternal asthma: in children whose mothers had asthma, the increase in FEF_50_ was greater and accompanied by increased FEV_1_ and FVC.^12^

From these studies, it remains unclear if the reported increase of volumes with breastfeeding involves different lung compartments proportionally. For instance, increased lung volumes might be explained by more alveoli or by larger alveoli due to structural differences or hyperinflation. Proportional increases in all lung volumes would suggest genuinely larger lungs, while increases limited to residual volume (RV), functional residual capacity (FRC) and total lung capacity (TLC) would suggest the presence of hyperinflation. We used the novel technique of ^3^He MR, which measures freedom of gas diffusion in alveolar-acinar compartments and provides a surrogate measure of alveolar size, to determine whether the reported increase in lung volume is associated with proportionately increased alveolar size (suggesting lung growth) or disproportionately increased alveolar size (suggesting hyperinflation). Using this technique, we recently reported evidence for continued alveolarisation throughout childhood and adolescence^13^ and evidence for catch-up alveolarisation in ex-preterm children.^14^ We have now reanalysed this data set to investigate the association between breastfeeding and plethysmographic lung volumes and alveolar dimensions. No previous studies have investigated the effect of breastfeeding on these measurements.

The aim of our study were thus to determine (1) whether plethysmographic lung volumes and alveolar size are associated with duration of breastfeeding, and (2) whether the potential association between breastfeeding and lung volumes differs depending on mother's history of asthma.

## Materials and methods

The study was approved by the Ethics Committee of the Leicestershire Health Authority and written consent had been obtained from all participants and their parents.

### Study population

For this study, we analysed a data set of 111 children and young adults (aged 11–21 years) from Leicestershire, UK, who had participated in a complex study on alveolar dimensions, measured by ^3^He MR, the ‘Helium study’.^13^ ^14^ The original study was not designed specifically for analysing the association with breastfeeding. We included for analysis participants who had been born at 32 weeks of gestation or later, had a birth weight of over 1500 g, who had no congenital anomalies or chronic lung diseases, such as cystic fibrosis or bronchopulmonary dysplasia, and in whom prospectively collected information on breastfeeding was available.

The participants had been recruited from the Leicester Respiratory Cohorts, consisting of two population-based cohorts based on random samples of children born in Leicestershire between 1987 and 1989 (the 1990 cohort), and between 1993 and 1997 (the 1998 cohort). The Leicestershire Health Authority Child Health Database, later called the Leicester Specialist Community Child Health Services Database, had been used to draw the sample and obtain baseline routine data.^15^ Parents of participating children received the first set of respiratory questionnaires in 1990 and 1998, respectively, when the children were 0–5 years old. The participants were then followed up repeatedly with questionnaires, every 2–4 years.

In total, 410 participants from the Leicester Respiratory Cohorts had been invited to the Helium study. A total of 125 (30.5%) participated, and we obtained ^3^HeMR measurements in 114 participants. Of the 114 with ^3^He MR measurements, 111 were eligible for the present study, based on criteria described above and availability of breastfeeding data.

### Health records and questionnaire data

We extracted perinatal data for all participants from Leicestershire Health Authority Child Health Database, including mother's ethnicity, birth weight and gestational age. We had assessed respiratory symptoms, and individual and family-related exposures initially in 1990 and 1998, and in several follow-up questionnaires in 2001, 2003 and 2006. At the time of the helium study, all participants and their parents were asked to complete a new questionnaire, providing information on health history, environmental exposures and demographic data.

Breastfeeding was defined as total duration of breastfeeding, regardless of exclusivity. We recorded duration of breastfeeding in the first questionnaire (applied at age 0–5 years). The question asked if the child had been breastfed and, if yes, for how long, with the following response options: *less than a month; 1–3 months; 4–6 months* and *more than 6 months*. Owing to the small sample size, we combined the responses into one of three categories: (1) *no breastfeeding,* (2) *breastfeeding for less than or equal to 3 months* and (3) *breastfeeding for more than 3 months*.

We considered as potential confounders those factors that might be a common cause of both exposure (duration of breastfeeding) and outcomes (lung and alveolar volumes) or be on the pathway of a common cause. For our analysis these included: Townsend score—an area-based deprivation score^16^—birth weight, preterm status, smoking during pregnancy, maternal asthma, maternal ethnicity and early-onset wheeze, described in more detail below. Participants were considered preterm if their gestational age, extracted from the birth registry, was under 37 weeks. Information on mother's history of smoking during pregnancy (*yes/no*) was collected in the initial questionnaire and updated at the time of measurements. Maternal history of asthma (*yes/no*) was self-reported based on the question: “Has the child's mother ever suffered from any of the following conditions :(…) wheezing; asthma?” We coded maternal asthma as ‘yes’ if the mother reported wheezing or asthma. Maternal ethnicity was extracted from birth records and categorised into *White* and *south-Asian*; the south-Asian group, which includes people of Indian/Sri Lanka, Pakistani or Bangladesh origin, is the largest ethnic minority in Leicestershire and one of the sampling strata of the Leicester Respiratory Cohorts. We defined *early-onset wheeze* as any wheezing with onset during the first year of life. *Early-onset wheeze* was included in the analysis in an attempt to control for a possible reverse causation, when early-onset wheeze, a possible precursor of future lung problems resulting in decreased lung function, might influence the duration of breastfeeding. In order to avoid over-adjustment, we did not include in the analysis variables that theoretically might be on the causal path, such as participant's history of asthma.^17^

### Physiological measurements

At the time of the study, height and weight were measured, and lung mechanics were assessed by performing spirometry and full-body plethysmography (Jaeger Masterscreen Body, Wuerzburg, Germany). The highest values of forced expired volume in 1 s (FEV_1_) and forced vital capacity (FVC) were reported, together with forced expiratory flow at 50% vital capacity (FEF_50_) from the manoeuvre with the largest sum of FEV_1_ and FVC. From plethysmography, the mean value of functional residual capacity (FRC), the largest vital capacity (VC), and the residual volume (RV) and total lung capacity (TLC) associated with the largest VC were reported. The measurements conformed to the American Thoracic Society/European Respiratory Society task force specifications,^18^ ^19^ and were reviewed for repeatability and quality control by a specialist respiratory physiologist (CSB).

To assess the average dimensions of the alveoli, we used the hyperpolarised ^3^Helium MR technique (^3^HeMR), which measures the restricted diffusion of hyperpolarised ^3^He in a constrained space such as the alveoli. The measurement provides a surrogate of alveolar dimension, the *apparent diffusion coefficient* (ADC). The participant is required to lie supine and at the end of a normal expiration, she/he inhales a bolus of 600 mL of a mixture of hyperpolarised ^3^He and ^4^He, and holds the breath for approximately 10 s. ^4^He is an abundant, naturally occurring non-radioactive helium isotope, while ^3^He is a rare non-radioactive isotope of helium that can be polarised.^20^ The MR measurements were performed with a 0.15 T permanent magnet system (Intermagnetics General Corporation, New York, New York, USA) using a modified rapid acquisition with refocused echoes (RARE) sequence (64 echoes, echo time=14 ms, acquisition time=896 ms, fixed gradient strength (b=0.3 s.cm^−2^), and slice select and phase gradients turned off).^13^ The technique is non-invasive and radiation-free, but expensive and used in only few centres worldwide. At least three technically satisfactory ADC values were obtained for each participant and the mean value was reported. We corrected ADC values for differences in ^3^He concentration and bolus size in relation to FRC.^13^ ^14^ The measurements were highly repeatable, with a within-subject coefficient of variation of 3.1%.^13^

### Statistical analysis

The association between duration of breastfeeding and lung volumes and alveolar size was analysed using multivariable linear regression. The outcomes of interest were spirometric measures (FVC, FEV_1_ and FEF_50_), plethysmographic measures (FRC, VC, RV and TLC), and alveolar size (ADC). To assess relative (percent) differences between levels of breastfeeding, we first log-transformed the outcomes, performed the regressions and then exponentiated estimated coefficients. Thus, the (exponentiated) regression constant and slope represent geometric means and relative differences, respectively. The analysis was performed in three steps: first we ran a model controlling only for variables needed to standardise lung function measurements, namely age, sex and height *(basic model)*. Second, we ran a model including potential confounders, namely preterm status, birth weight, Townsend score, smoking during pregnancy, maternal asthma, maternal ethnicity and early-onset wheeze *(adjusted model);* third, we ran a model in which we added an interaction term between breastfeeding and maternal asthma to test for effect modification by maternal asthma *(effect-modification model)*. The analyses of ADC were adjusted, additionally, for the natural logarithm of FRC, because of an expected increase in ADC with FRC.^13^ Thus, regression coefficients for the ADC models reflect relative differences in ADC over and above those accounted for by changes in FRC due to lung growth. These relative differences in ADC due to the risk factors in the regression model are also expressed in terms of estimated differences in mean alveolar volume using the relationship ADC ratio=(volume ratio)^0.415^ derived in Narayanan *et al*.^13^

Based on the third model with an interaction term between breastfeeding and maternal asthma, we calculated the means of the outcome measures for children of mothers with and without asthma. All analyses were performed using Stata V.12 (Stata Corporation, Austin, Texas, USA).

## Results

### Sample characteristics

Detailed data on sample characteristics are presented (table 1). Among participants 24 (22%) had never been breastfed, 37 (33%) had been breastfed 3 months or less, and 50 (45%) had been breastfed over 3 months; the mothers of 22 (20%) reported having asthma. Girls, children of mothers without asthma and of mothers who did not smoke during pregnancy tended to be breastfed longer. We did not find evidence for differences in spirometry or plethysmographic measurements and ADC between categories of breastfeeding.

**Table 1:** Characteristics of study population, by duration of breastfeeding (N=151)

	Breastfeeding categories				
	Total (N=111)	Never (N=24)	≤3 months (N=37)	>3 months (N=50)	p Value*
Age† (years)	14.1 (2.5)	14.7 (2.7)	14.3 (2.9)	13.5 (2.0)	0.111
Height† (cm)	160.8 (10.9)	164.0 (13.5)	158.7 (10.8)	160.9 (9.4)	0.190
Weight† (kg)	53.8 (13.1)	58.2 (17.2)	52.8 (13.1)	52.5 (10.5)	0.189
Gestational age† (weeks)	38.5 (2.3)	39.0 (1.9)	38.4 (2.5)	38.9 (2.0)	0.371
Birth weight (g)	3235.8 (599.2)	3111.2 (622.2)	3212.7 (605.1)	3313.8 (583.9)	0.386
Sex‡					
Male	51 (46.0)	23 (63.9)	14 (32.6)	35 (48.1)	0.021
Female	60 (54.0)	13 (36.1)	29 (67.4)	37 (51.4)	
Ethnicity‡					
South-Asian	24 (21.6)	5 (20.8)	9 (37.5)	10 (41.7)	0.884
White	87 (78.4)	19 (21.8)	28 (32.2)	40 (45.9)	
Smoking during pregnancy‡					
No	100 (90.1)	18 (18.0)	36 (36.0)	46 (46.0)	0.014
Yes	11 (9.9)	6 (54.5)	1 (9.1)	4 (36.4)	
Early-onset wheeze‡					
No	88 (79.3)	19 (21.6)	31 (35.2)	38 (43.2)	0.676
Yes	23 (20.7)	5 (21.7)	6 (26.1)	12 (52.2)	
Maternal asthma‡					
No	88 (79.3)	15 (17.05)	31 (35.23)	42 (47.73)	0.052
Yes	22 (19.8)	9 (40.91)	6 (27.27)	7 (31.82)	
Missing	1 (0.9)	0 (0.0)	0 (0.0)	1 (100.0)	
FVC§ (L)	3.5 (0.7)	3.76 (0.92)	3.41 (0.74)	3.53 (0.63)	0.183
FEV_1_§ (L)	3.0 (0.6)	3.23 (0.75)	2.94 (0.62)	3.04 (0.52)	0.197
FEF_50_§ (L/s)	3.6 (0.6)	3.78 (0.74)	3.50 (0.62)	3.61 (0.53)	0.220
FRC§ (L)	2.2 (0.5)	2.37 (0.59)	2.17 (0.51)	2.19 (0.40)	0.227
RV§ (L)	1.2 (0.2)	1.31 (0.29)	1.22 (0.25)	1.22 (0.19)	0.233
TLC§ (L)	4.7 (0.9)	4.94 (1.15)	4.50 (0.94)	4.64 (0.78)	0.196
ADC¶ (cm^2^/s)	0.096 (0.012)	0.095 (0.012)	0.096 (0.012)	0.095 (0.013)	0.444

*The p value is based on a χ^2^ test.

†Mean (SD).

‡N (%); the percentages represent breastfeeding frequencies within levels of confounder (row percentages).

§The means of the outcome variables are predicted values adjusted for age, sex, height, weight using linear regression.

¶The ADC analysis was performed using the natural logarithm, adjusting for age, sex, height and ln FRC; the estimates were back-transformed from the logarithmic scale to the original scale, therefore the estimated means are geometric means.

ADC, apparent diffusion coefficient; ETS, exposure to tobacco smoke; FEF_50_, forced mid-expiratory flow; FEV_1_, forced expiratory volume at 1 s; FRC, functional residual capacity; FVC, forced vital capacity; RV, residual volume; TLC, total lung capacity; VC, vital capacity.

### Breastfeeding and lung volumes

#### Basic and adjusted model

Table 2 presents the results of the basic and adjusted models. When compared with participants who had not been breastfed, the TLC of participants breastfed >3 months was on average larger by over 6% in the basic and adjusted model. We found no strong evidence for an association between duration of breastfeeding and any of the other lung function outcomes, regardless of adjustment for potential confounders. Although participants breastfed for >3 months tended to have larger lung volumes and flows than non-breastfed participants, 95% CIs (95% CI) for differences included 0 and the p values were all >0.1.

**Table 2:** Association between breastfeeding and lung function measurements in all participants, basic model and fully adjusted model*

Lung function (unit)	Basic model†		Adjusted model‡	
	estimates (CI)	p Value	estimates (CI)	p Value
FVC§ (L)				
No BF (mean)	3.31 (3.12 to 3.49)		3.39 (3.18 to 3.61)	
BF ≤3 months (% difference)	3.07 (−4.30 to 10.44)	0.407	4.20 (−3.03 to 11.43)	0.245
BF >3 months (% difference)	4.44 (−2.65 to 11.52)	0.210	4.20 (−2.40 to 10.80)	0.203
FEV_1_§ (L)				
No BF (mean)	2.82 (2.66 to 2.98)		2.93 (2.73 to 3.13)	
BF ≤3 months (% difference)	4.33 (−3.43 to 12.08)	0.264	5.42 (−2.34 to 13.17)	0.160
BF >3 months (% difference)	5.66 (−1.80 to 13.12)	0.126	5.41 (−1.67 to 12.48)	0.124
FEF_50_§ (L/s)				
No BF (mean)	3.20 (2.86 to 3.55)		3.46 (2.98 to 3.95)	
BF ≤3 months (% difference)	9.16 (−6.04 to 24.35)	0.217	9.71 (−7.14 to 26.56)	0.237
BF >3 months (% difference)	9.92 (−4.54 to 24.39)	0.159	9.67 (−5.62 to 24.95)	0.194
FRC§ (L)				
No BF (mean)	1.99 (1.87 to 2.12)		1.96 (1.79 to 2.13)	
BF ≤3 months (% difference)	3.03 (−5.57 to 11.64)	0.483	4.72 (−5.12 to 14.55)	0.336
BF >3 months (% difference)	6.58 (−1.89 to 15.06)	0.116	5.76 (−3.36 to 14.87)	0.203
RV§ (L)				
No BF (mean)	1.11 (1.01 to 1.21)		1.08 (0.96 to 1.20)	
BF ≤3 months (% difference)	−1.27 (−12.62 to 10.09)	0.828	−0.59 (−12.75 to 11.57)	0.924
BF >3 months (% difference)	8.32 (−3.53 to 20.18)	0.152	11.27 (−1.22 to 23.75)	0.062
TLC§ (L)				
No BF (mean)	4.31 (4.11 to 4.51)		4.38 (4.15 to 4.62)	
BF ≤3 months (% difference)	3.00 (−3.13 to 9.13)	0.330	4.36 (−1.59 to 10.31)	0.143
BF >3 months (% difference)	6.13 (0.12 to 12.14)	0.039	6.57 (0.99 to 12.14)	0.017
ADC§ (cm^2^/s)				
No BF (mean)	0.09 (0.09 to 0.10)		0.09 (0.08 to 0.10)	
BF ≤3 months (% difference)				
Absolute ADC value	3.00 (−3.58 to 9.57)	0.365	0.59 (−6.31 to 7.49)	0.867
Average alveolar size¶	7.38 (−8.45 to 24.71)		1.45 (−14.51 to 19.03)	
BF >3 months (% difference)				
Absolute ADC value	0.69 (−5.43 to 6.81)	0.824	1.88 (−4.56 to 8.32)	0.563
Average alveolar size¶	1.69 (−12.52 to 17.17)		4.64 (−10.72 to 21.18)	

*The analyses were performed using the natural logarithm of the outcome variable; the estimates were back-transformed from the logarithmic scale to the original scale, therefore the means represent the geometric means, not the arithmetic ones.

†In the basic model we adjusted for age, sex and height.

‡In the adjusted model we included, additionally*, preterm status, birth weight, Townsend score, smoking during pregnancy, maternal asthma, maternal ethnicity* and *early-onset wheeze* (wheezing history with onset during the first year of life).

§All analysis were performed using the natural logarithm; the estimates were back-transformed from the logarithmic scale to the original scale, therefore the coefficients are multiplicative (they represent ratios; eg, in the basic model, participants breastfed ≤3 months had an ADC absolute value 3% higher than participants who were not breastfed).

¶Calculated with the formula ADC ratio=(volume ratio)^0.415^, see Narayanan *et al*.^13^

ADC, apparent diffusion coefficient; BF, breastfeeding; FEF_50_, forced mid-expiratory flow; FEV_1_, forced expiratory volume at 1 s; FRC, functional residual capacity; FVC, forced vital capacity; RV, residual volume; TLC, total lung capacity.

#### Effect-modification model

**Figure 1:**
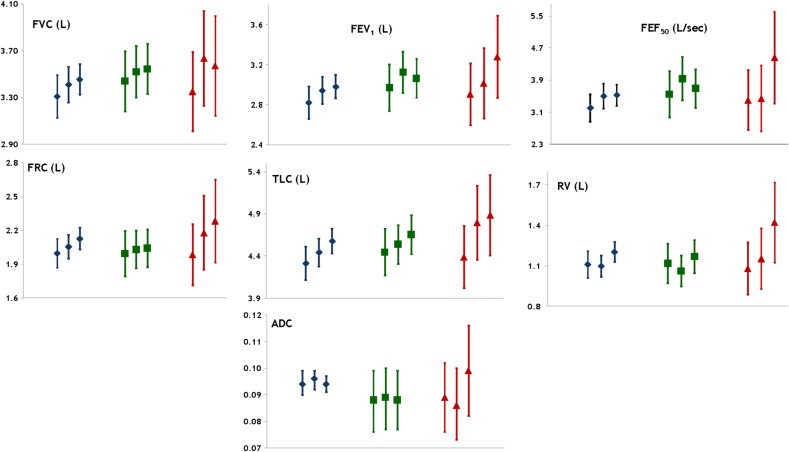
Association between breastfeeding and lung function measurements by maternal asthma, fully adjusted model with interaction. The graph represents the adjusted means and CIs for each breastfeeding category (from left to right: none, ≤3 months and >3 months), in the entire sample (blue diamonds) and stratified by children of mothers with no asthma (green squares) and children of mothers with asthma (red triangles). The estimates come from the adjusted model with interaction.

The results from the model that tested an effect modification by maternal asthma are presented in table 3 and figure 1. We did not find significant evidence for an effect modification by maternal asthma. Among participants born to mothers with asthma there was a tendency towards larger lung volumes and alveolar size in those who were breastfed >3 months compared with those who were not breastfed but in offspring of mothers without asthma these differences were close to zero. When compared with participants who had not been breastfed, the FRC of participants breastfed >3 months was on average larger by 2.4% in offspring of non-asthmatic mothers but larger by 15.2% in those born to asthmatic mothers. Similar differences between children of asthmatic and non-asthmatic mothers were found for the other lung volumes (table 3). The ADC of participants breastfed over 3 months was 0.5% lower in those born to non-asthmatic mothers but 11.0% higher in those born to asthmatic mothers. Using the formula ADC ratio=(volume ratio)^0.415^, we determined that among participants breastfed for over 3 months the average alveolar size was smaller by 1.2% in participants of non-asthmatic mothers but 28.6% larger in participants of asthmatic mothers compared to participants who were not breastfed (table 3).

**Table 3:** Association between breastfeeding and lung function measurements by maternal asthma, fully adjusted model with interaction

Lung function (unit)	Mothers without asthma	Mothers with asthma	
	*estimate (CI)*	*estimate (CI)*	*p-interaction*
FVC* (L)			
No BF (mean)	3.44 (3.18 to 3.70)	3.35 (3.01 to 3.69)	
BF ≤3 months (% difference)	2.38 (– 6.37 to 11.13)	8.54 (– 5.68 to 22.75)	0.471
BF >3 months (% difference)	3.09 (– 4.93 to 11.10)	6.59 (– 7.92 to 21.09)	0.689
FEV_1_* (L)			
No BF (mean)	2.97 (2.73 to 3.20)	2.90 (2.59 to 3.21)	
BF ≤3 months (% difference)	5.27 (– 4.18 to 14.71)	3.80 (– 10.48 to 18.07)	0.869
BF >3 months (% difference)	3.23 (– 5.19 to 11.65)	12.88 (– 3.25 to 29.01)	0.308
FEF_50_* (L/s)			
No BF (mean)	3.54 (2.97 to 4.12)	3.40 (2.66 to 4.14)	
BF ≤3 months (% difference)	11.10 (– 9.47 to 31.66)	1.15 (– 27.30 to 29.61)	0.591
BF >3 months (% difference)	4.20 (– 13.21 to 21.60)	31.27 (– 7.11 to 69.65)	0.198
FRC* (L)			
No BF (mean)	1.99 (1.79 to 2.20)	1.98 (1.71 to 2.26)	
BF ≤3 months (% difference)	1.87 (– 9.90 to 13.64)	9.90 (– 9.60 to 29.40)	0.490
BF >3 months (% difference)	2.45 (– 8.35 to 13.24)	15.20 (– 6.01 to 36.40)	0.299
RV* (L)			
No BF (mean)	1.12 (0.97 to 1.26)	1.08 (0.89 to 1.27)	
BF ≤3 months (% difference)	−4.98 (– 19.15 to 9.18)	6.67 (– 17.75 to 31.10)	0.414
BF >3 months (% difference)	4.73 (– 9.51 to 18.97)	31.65 (0.36 to 62.93)	0.117
TLC* (L)			
No BF (mean)	4.44 (4.17 to 4.72)	4.38 (4.02 to 4.75)	
BF ≤3 months (% difference)	2.07 (– 5.08 to 9.23)	9.32 (– 2.45 to 21.08)	0.303
BF >3 months (% difference)	4.65 (– 2.04 to 11.34)	11.36 (– 1.08 to 23.80)	0.365
ADC* (cm^2^/s)			
No BF (mean)	0.09 (0.08 to 0.10)	0.09 (0.08 to 0.10)	
BF ≤3 months (% difference)			
absolute ADC value	1.41 (– 6.87 to 9.70)	−4.00 (– 16.12 to 8.13)	0.437
average alveolar size†	3.41 (– 15.82 to 24.98)	1.69 (– 12.52 to 17.17)	
BF >3 months (% difference)			
absolute ADC value	−0.47 (– 7.89 to 6.96)	11.04 (– 3.61 to 25.70)	0.174
average alveolar size†	−1.20 (– 17.98 to 17.70)	28.58 (– 8.45 to 73.50)	

In this model we adjusted for *age, sex, height, preterm status, birth weight, Townsend score, smoking during pregnancy, maternal asthma, maternal ethnicity,* and *early-onset wheeze* (wheezing history with onset during the first year of life) and included an interaction term between *breastfeeding* and *maternal asthma.* The means and coefficients for the groups of children of mother with and without asthma were calculated using the regression coefficients for breastfeeding, maternal asthma and the interaction term, using the command lincom in Stata, which computes point estimates, CIs and p-values for linear combinations of coefficients.

*All analyses were performed using the natural logarithm; the estimates were back-transformed from the logarithmic scale to the original scale, therefore the coefficients are multiplicative (they represent ratios; eg, in children of mothers without asthma, participants breastfed ≤3 months had an ADC value 1.4% higher than participants who were not breastfed)

†The average alveolar size was calculated with the formula *ADC ratio=(volume ratio)*^*0.415*^*,* see Narayanan et al.^13^

ADC, apparent diffusion coefficient; BF, breastfeeding; FEF_50_, forced mid-expiratory flow; FEV1, forced expiratory volume at 1 second; FRC, functional residual capacity; FVC, forced vital capacity; p-int, p-interaction; RV, residual volume; TLC, total lung capacity.

## Discussion

### Findings and interpretation

In this study we did not find evidence of an association between breastfeeding duration and lung volumes or alveolar dimensions at school age, except for larger TLC values in children breastfed over 3 months. However, we observed a consistent trend towards larger alveoli and larger lung volumes, both spirometric and plethysmographic, in children of asthmatic mothers who had been breastfed over 3 months compared to those not breastfed. While the findings were comparable to previous reports,^12^ they did not reach statistical significance for any of the tested outcomes in this small study.

Several authors have found positive associations between breastfeeding and lung function in school-age children.^6–11^ We have reported previously in a larger study that breastfed children had increased FEF_50_ compared with non-breastfed children. This increase was larger in participants born to mothers with asthma, with evidence for a dose–response relationship with duration of breastfeeding. Furthermore, it was accompanied by increases in FVC and FEV_1_. In the present study we found differences that were similar or larger in magnitude than in our earlier study, but they failed to reach statistical significance. This is not surprising as the two studies are not independent: 81% of the children in the present study were also included in the previous study. However, because of the costly and time consuming nature of ^3^HeMR measurements, the sample size in the present study was more modest and thus, the statistical power reduced.

Our results were suggestive of an increase in all volumes associated with breastfeeding in children of asthmatic mothers. The precision of our estimates was too small to distinguish between the hypotheses of congruent volume increases in all components versus hyperinflation. The relative increases were largest for mean alveolar volume, suggesting that volume increases were not accompanied by a proportional increase in alveolar number.

If we consider that these findings do reflect real differences in the population, it is difficult to speculate why this might be so, and why this difference is seen only in children of mothers with asthma. A possible explanation is that there were influences associated with secretion of lung growth factors in breast milk of asthmatic mothers. Another possible explanation could be residual confounding by severity of maternal asthma; perhaps mothers with more severe asthma were less likely to breastfeed, but more likely to have children with low lung volumes. Although the analysis adjusted for presence of maternal asthma, we did not have information on asthma severity in the mother.

### Strengths and limitations

A strength of this study is the large range of lung measurements, including spirometry and plethysmography and in vivo measurements reflecting alveolar dimension.^13^ ^14^ Furthermore, the study was able to consider important potential confounding including early onset of wheeze, through which we attempted to control for possible reverse causation.^12^

The main limitation is the small sample size which was due to the complexity and cost of the techniques involved. While the study had been adequately powered to assess potential age-related changes of alveolar dimensions 13 and differences between term and preterm children,^14^ it was probably too small to detect minor differences resulting from duration of breastfeeding in such a distal outcome as lung function at school age. This was further complicated by the attempt to determine if the association is influenced by maternal asthma, performing an analysis with an interaction term; there were only seven participants of mothers with asthma who had been breastfed over 3 months (table 1).

Another limitation is a possible recall bias in reporting duration of breastfeeding. The participants reported duration of breastfeeding when the children were 1–4 years old. However, the question had shown an excellent short-term repeatability in our cohort, with a Cohen's κ of 0.96 21 and there is independent evidence suggesting that long-term recall of breastfeeding is excellent.^22^

Diffusion-weighted ^3^HeMR uses the degree of restriction to diffusion of ^3^He as a proxy for dimensions of the enclosing structure. It follows that the diffusion displacement, s, should be of a similar order of magnitude to the distance between the barriers. If ‘s’ is too small, the 3-He molecules are not restricted by the barriers and ADC approximates free diffusion coefficient, D. If ‘s’ is too large, it is affected by the structures outside the barriers. Parra-Robles *et al*^23^ contended that the diffusion time employed in this study would result in ‘s’ that would be sensitive to structures outside the alveoli. However, ‘s’ in our case is only 1.58 times larger than they suggested (because of the square root relationship)^24^ Also, while it was true that some of the ^3^He atoms in our study do sample the space outside an individual alveolus and may move to the alveolar duct space, the measurements still reflect alveolar dimensions as the alveolar duct does not have an independent wall. The ultrastructure of the periphery of the lung is made up of alveolar septae. As long as the alveolar duct dimension does not increase or decrease independent of alveolar dimensions, our ADC measurements are valid proxies of alveolar dimensions. This is explored in further detail in our reply^24^ to Parra-Robles *et al*^23^

The relationship between ADC and volume was derived by measuring ADC in children at different levels of inflation.^13^ Using this relationship to extrapolate alveolar volume ratio between participants from ADC ratio assumes similar alveolar geometry across participants, and therefore alveolar volume ratio should be interpreted with caution.

A hypothesis that could not be explored in this study is that a possible association of breastfeeding with lung function measurement is age dependent, that is, the association might be present (or stronger) at younger age, compared with older ages. Unfortunately, despite the large age range of our participants, the size of the sample against which we could test this hypothesis is too small; for example, only five participants were 10 years or younger.

While not providing a definite answer to the research question, the study offered hints that in children of mothers with asthma, those who are breastfed might have larger lung volumes, and it opened the path towards investigating the possible mechanisms involved. Further investigations with larger sample sizes are essential to answer this important question. Although the differences in lung volumes and alveolar size might be small for individual children, if these are confirmed in a larger study it would have important consequences for public health since the proportion of women with asthma is high.
